# Species Distribution Model for the Asian Tapir and Vegetation Characteristics of Batang Gadis National Park, North Sumatra, Indonesia

**DOI:** 10.21315/tlsr2023.34.2.4

**Published:** 2023-07-21

**Authors:** Wanda Kuswanda, Freddy Jontara Hutapea, Muhammad Hadi Saputra, Bobby Nopandry

**Affiliations:** 1 Research Organization for Life Sciences and Environment, National Research and Innovation Agency (BRIN), Gedung B.J. Habibie, Jl. M.H. Thamrin No. 8, Central Jakarta 10340, DKI Jakarta, Indonesia; 2 School of Ecosystem and Forest Sciences, Faculty of Science, The University of Melbourne, Creswick, Victoria 3363, Australia; 3 Batang Gadis National Park Institute, Directorate General of Nature Resources and Ecosystem, Ministry of Environment and Forestry, Indonesia

**Keywords:** Asian Tapir, Species Distribution Model, Vegetation Characteristics, Batang Gadis National Park, Feed Plants

## Abstract

The Asian tapir is a primitive mammal whose habitat is heavily fragmented due to human activities. Studies on the Asian tapirs in Sumatra are very few, thereby basic information for developing tapir conservation programmes is limited. This study aimed to develop the species distribution model to map the potential distribution of tapirs in Batang Gadis National Park (BGNP), investigate the characteristic of tapir habitat, and identify tapir feed plants around BGNP. The model was developed using the Maximum Entropy (Maxent) approach, based on the existing information on tapir occurrence in BGNP and environmental variables. Vegetation characteristics in different land cover (primary forests, secondary forests, and open fields) were investigated using the strip transect method. This study found that zonal classification, temperature and precipitation have the greatest percentage contribution to the model. The model estimated that around 24.45% of BGNP areas are suitable for tapir habitat, and tapirs distribute near community gardens. Our results also showed that plant diversity at study sites was categorised as moderate-high. About 23 plant species dominated by the *Moraceae* family were identified as feed plants for tapirs. In developing tapir conservation programmes, BGNP management needs to consider tapir distribution that is closed to community gardens. We propose BGNP to enrich feed plants in open fields of the wilderness and traditional zones; reduce the canopy cover in the wilderness and utilisation zones to stimulate the growth of feed plants; facilitate local people to live harmoniously with tapirs; involve local communities in tapir conservation programmes; encourage local communities to plant non-palatable crops for tapirs; and provide a compensation scheme.

**Highlights**

- About 24.45% of Batang Gadis National Park (BGNP) areas are suitable for tapir habitat.
- Tapirs distribute near the traditional zone where the community garden islocated.
- About 23 tapir feed plant species dominated by *Moraceae* family were observed in BGNP.

## INTRODUCTION

Tapirs, the most primitive large-bodied mammal, have inhabited tropical forests since the Pleistocene period (Samantha *et al*. 2020). The Asian tapir (*Tapirus indicus*) is one of the four remaining species of tapirs distributed in several countries of Southeast Asia such as Indonesia, Laos, Malaysia, Myanmar, Thailand and Vietnam (Novarino 2005; O’farrill *et al*. 2013). In Indonesia, tapirs can be found in almost all provinces of Sumatra Island (Holden *et al*. 2003; Marlius *et al*. 2018). Tapirs are known as solitary and nocturnal animals (Marlius *et al*. 2018). Tapirs have important ecological roles as seed dispersers and seed predators (Cranbrook & Piper 2009; Wittayarat *et al*. 2021). The seed dispersed by tapirs may grow and facilitate carbon sequestration (Paolocci *et al*. 2019).

In recent decades, the population of tapirs declined dramatically due to habitat destruction, poaching, road construction, and global warming (Meyer *et al*. 2022; Samantha *et al*. 2020). The remaining tapir population in nature is below 2,500 individuals, and it is projected to decrease by 20% in 2040 (Wittayarat *et al*. 2021). The Asian tapir is currently classified as an endangered species by the International Union for Conservation of Nature (IUCN) and listed in Appendix A of the Convention on International Trade in Endangered Species (CITES) (Traeholt *et al*. 2016). In Indonesia, the Asian tapir has been protected since 1931 (Holden *et al*. 2003).

Batang Gadis National Park (BGNP) is one of the most important tapir habitats on Sumatra Island. Kuswanda and Mukhtar (2010) found that the density of tapirs in this national park is about 0.09 individuals/ha. Meanwhile, Linkie *et al*. (2013) noted a high probability of occurrence of tapirs in this national park (0.90 ± 0.06). However, some concerns about the existence of tapirs in BGNP still exist, notably due to the land use change and habitat destruction. In 2012, about 30% of BGNP was excised for gold mining (Linkie *et al*. 2013). Studies related to tapirs in Sumatra, especially in BGNP, are still limited. This condition has some consequences for the establishment of tapir conservation programmes in BGNP.

Schank *et al*. (2015) pointed out that the species distribution model is the foundation to understand the ecology, conservation, and management strategies of a species. Currently, this model has been developed for various purposes, especially for conservation management. The species distribution model quantifies the probability of species distribution in certain areas and times using environmental variables (Franklin 2010). This model is generally known as species distribution modeling (SDM) and is described formally as Niche modeling, habitat modeling, habitat suitability modelling, and predictive habitat distribution modelling (Miller 2010). The most common approach used to develop SDM is maximum entropy (Maxent). Maxent models suitable environmental conditions of a species based on species occurrences data and a set of environmental variables (Phillips *et al*. 2006). Maxent is generally easy to use and efficient to identify suitable areas for a species (Clements *et al*. 2012; Cordeiro *et al*. 2016; Merow *et al*. 2013). Currently, SDM for tapir has been developed in other parts of the world, e.g., South America, Ecuador, Peninsular Malaysia and Peru (Cordeiro *et al*. 2016; More *et al*. 2022; Ortega-Andrade *et al*. 2015). However, this model is not developed yet for tapirs in BGNP.

The main objective of this study is to:

1. Develop the species distribution model to provide the potential distribution map of tapirs in BGNP.
2. Investigate the characteristic of tapir habitat.
3. Identify tapir feed plants around BGNP.

This study is expected to provide important information for tapir conservation programmes in BGNP.

## MATERIALS AND METHODS

### Study Site

**Figure 1:**
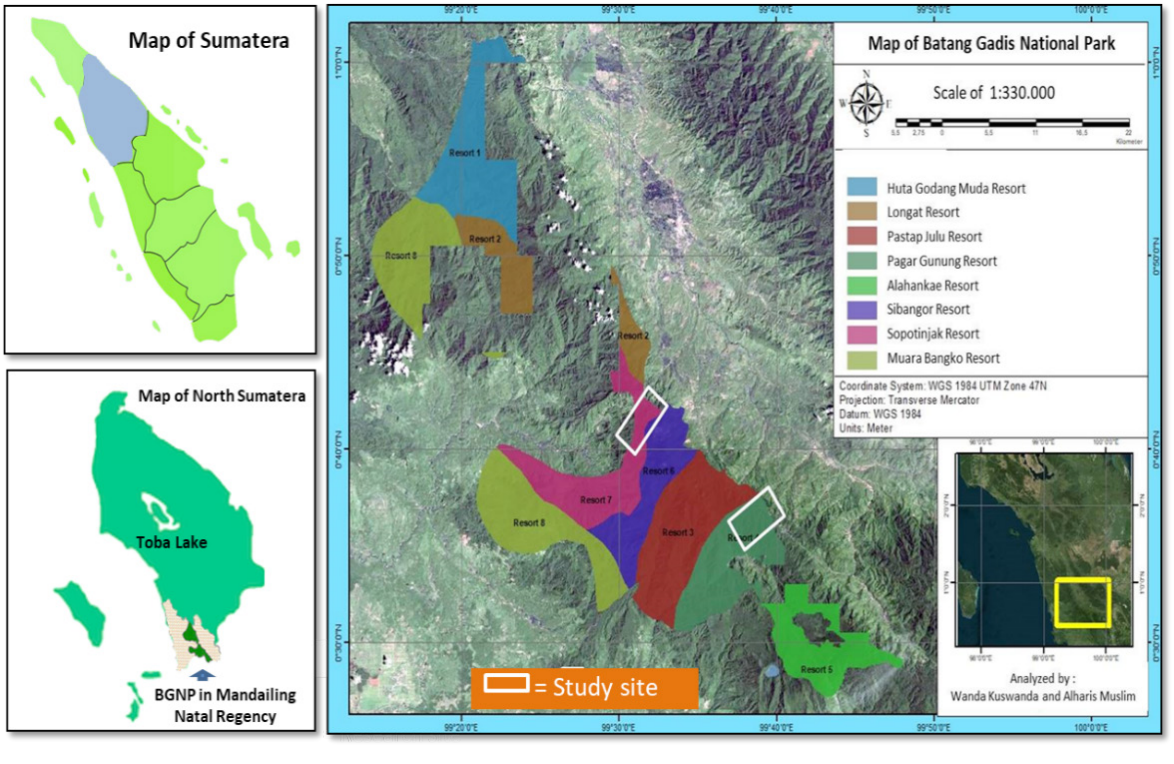
Study sites at BGNP (Source: Taman Nasional Batang Gadis).

This study was conducted in BGNP in Mandailing Natal (Madina) Regency (Fig. 1). Batang Gadis was designated as a national park on 29 April 2004 by the decree of the Minister of Forestry No: SK.126/Menhut-II/2004 on 29 April 2014 (Ministry of Forestry 2004). The BGNP originally covered about 108,000 ha of forests in the District of Mandailing Natal (Madina), North Sumatra Province. In 2012, the total area of the BGNP shrank to 72,804 ha (Decree of the Minister of Forestry: SK.3973/Menhut-VII/KUH/2014) (Ministry of Forestry 2014). The BGNP covers 10 districts and 32 villages. The BGNP comprises five zones, three management units, and eight resorts (Balai Taman Nasional Batang Gadis 2020).

Geographically, the BGNP is located between 99°12’45” and 99°47’45” East and between 0°27’15” and 1°01’57” North at an altitude between 300 m and 2,145 m (Kartawinata *et al*. 2004; Balai Taman Nasional Batang Gadis 2020). The annual rainfall in the BGNP is between 1,900 and 2,800 mm (Wibisono *et al*. 2009). The BGNP is high in biodiversity. Rambe *et al*. (2021) pointed out that the BGNP is inhabited by numerous wildlife, including Sumatran tiger (*Panthera tigris sumatrae*), forest goat (*Naemorhedus sumatrensis*), tapir (*T. indicus*), sun bear (*Helarctos malayanus*), sambar deer (*Cervus unicolor*), Indian muntjak (*Muntiacus muntjac*), pig-tailed macaques (*Macaca nemestrina*), clouded leopard (*Neofolis nebulosa*), golden cat (*Catophama teminckii*). The BGNP is also occupied by no more than 247 bird species, and 240 identified plant species (0.9% of plant species grow in Indonesia) (Nasution *et al*. 2018; Perbatakusuma *et al*. 2009).

### Data Collection

#### SDM development

Maxent uses several data inputs (spatial data with the same resolution and extent) to quantify the potential distribution of tapirs in BGNP. About 25 tapir occurrences derived from the Spatial Monitoring and Reporting Tool (SMART) patrol system in BGNP were used as data inputs to develop the model. The SMART patrol system is a tool developed to measure, evaluate, and improve monitoring in conservation areas (Sadikin *et al*. 2016). Environmental variables (Table 1) were used as the main predictors. The elevation data were collected from the Advanced Spaceborne Thermal Emission and Reflection Radiometer Global Digital Elevation Model (ASTER GDEM). The elevation map was created from the GDEM using ArcMap version 10. The slope was calculated from the Digital Elevation Model (DEM) (Austin 2007; Saputra & Lee 2021).

Climate data were collected from the WorldClim dataset that is providing several bioclimatic predictors for supporting ecological applications (O’Donnell & Ignizio 2012). The bioclimatic predictors used to develop the model are provided in Table 2. During the first run, we found that Bio 4, Bio 7 and Bio 15 did not have significant contributions to the model. Therefore, they are excluded from the final model.

BGNP was divided into five zones: core zone, specific zone, utility zone, wilderness zone and traditional zone (Balai Taman Nasional Batang Gadis 2020) (Table 3).

Land cover data were derived from satellite images in 2020. The land cover classification was modified from the Ministry of Environment and Forestry Land Use Land Cover Map Classification. In this study, the land cover in BGNP was classified into crops, forests, open areas, residential areas and shrubs (Table 4).

#### The characteristic of the tapir habitat

The investigation of the characteristic of tapir habitat and tapir feed plants was performed in Pagar Gunung Resort (Resort IV), Sopotinjak Resort (Resort VII), and their supporting villages between March and October 2021 (Fig. 1). Pagar Gunung and Sopotinjak Resorts are located at altitudes of 1,400 m and 1,200 m, respectively. Prabuningrum (2020) noted that these resorts are the main habitat for tapir in the BGNP.

**Figure 2:**
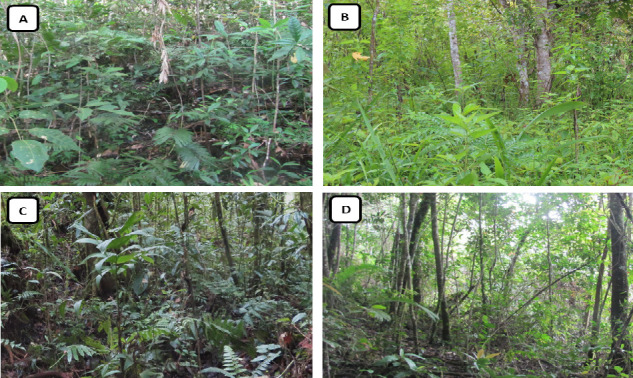
General pictures of land cover in study sites: (A) PFs in Sopotinjak Resort; (B) OFs in Sopotinjak Resort; (C) PFs in Pagar Gunung Resort; and (D) SFs in Pagar Gunung Resort.

**Figure 3:**
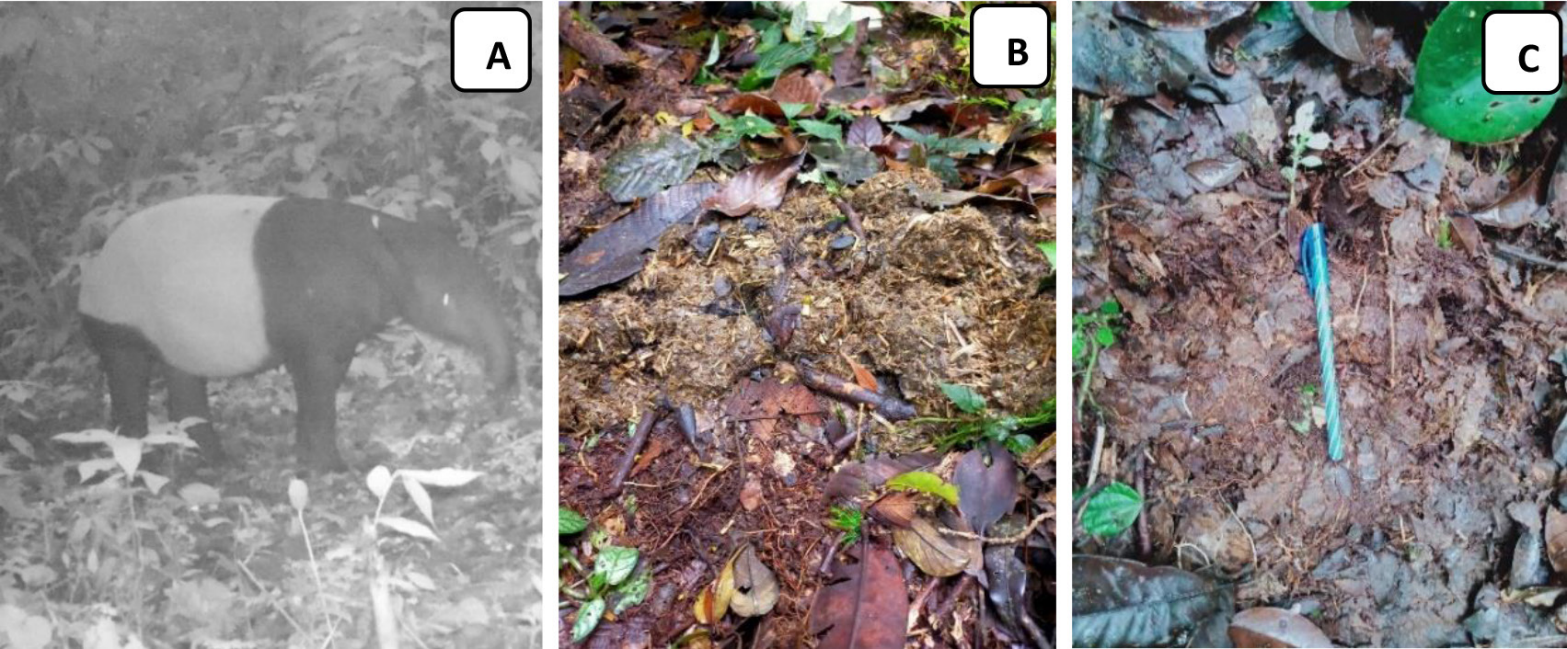
The spots identified as tapir habitat through (A) camera traps; (B) faeces; and (C) foot marks (Source: Taman Nasional Batang Gadis and this study).

Tapir habitat and feed identification were conducted in three different land covers: primary forests (PFs), secondary forests (SFs) and open fields (OFs) (Fig. 2). This study was performed in the spot which was detected by camera traps as tapir habitat (Fig. 3), or spots where tapir footmarks or faeces were observed during the study. Vegetation analysis was performed using the strip transect method with systematic random sampling. A 1.6 km line transect was installed in each land cover. Nested plots (2 × 2 m^2^ and 5 × 5 m^2^ plots) were then installed along the transect to record seedling and understory and sapling, respectively. The distance between plots was about 40 m (Alatar *et al*. 2012).

Vegetation analysis was confined to the sapling, seedling and understory as tapirs just used vegetation at these growth stages for food (Meyer *et al*. 2022; O’farrill *et al*. 2013; Santiapillai & Ramono 1990). The sapling is categorised as a plant with a stem circumference between 6.3 cm and 31.4 cm. The seedling is a plant with a stem circumference of less than 6.3 cm and/or heights of less than 1.5 m. The understory is a non-woody plant e.g., grass, shrubs and herbs (Fachrul 2007). Tapir feed plants were recorded during the vegetation analysis. Feed plants identification was performed by BGNP officers and local people who had a good knowledge of tapirs.

### Data Analysis

#### SDM for tapir

The spatial analysis was performed to provide a suitable area for tapir distribution across the BGNP. The data of tapir occurrence and environmental variables were converted into spatial data with the same extent and resolution to produce better results in the Maxent model. The resolution used in this study is 30 m. The coordinates for the data raster were extended to 0.45°N–1.02°N and 99.24°E –99.8°E.

The spatial analysis was conducted to produce a tapir potential distribution map. The probability of tapir distribution is arranged from 0 to 1, where a higher probability number indicates a higher probability of tapir distribution (Loi 2008). The probability threshold for an area classified as suitable for tapir distribution is greater than 0.4. The probability distribution < 0.4 was classified as an unsuitable area for tapir distribution.

The model was validated using the receiver operating characteristic (ROC) curve. The ROC curve analyses the performance of a model at all classification thresholds (Phillips *et al*. 2004). The quality of the model is determined by the Area Under Curve (AUC) values (Phillips *et al*. 2006). The higher the AUC values, the better the quality of the model is. A good model should have AUC values above 0.5 (Elith *et al*. 2006).

#### Tapir habitat characteristic analysis

The characteristics of vegetation were analysed using the species diversity index referring to the Shannon and Weaver equation (Shannon 1948) and the Hill index (Geng *et al*. 2019). Plant composition was analysed using the important value index (IVI) equation (Fachrul 2007; Tabares *et al*. 2018). The relationship between vegetation characteristics and plants consumed by the tapir was analysed using percentage analysis and Chi-square analysis with the Statistical Package for Social Science (SPSS) 23.0 software for Windows (IBM Corp. SPSS Statistics; Somers, NY, USA).

## RESULTS AND DISCUSSION

### SDM for Tapir in BGNP

**Figure 4:**
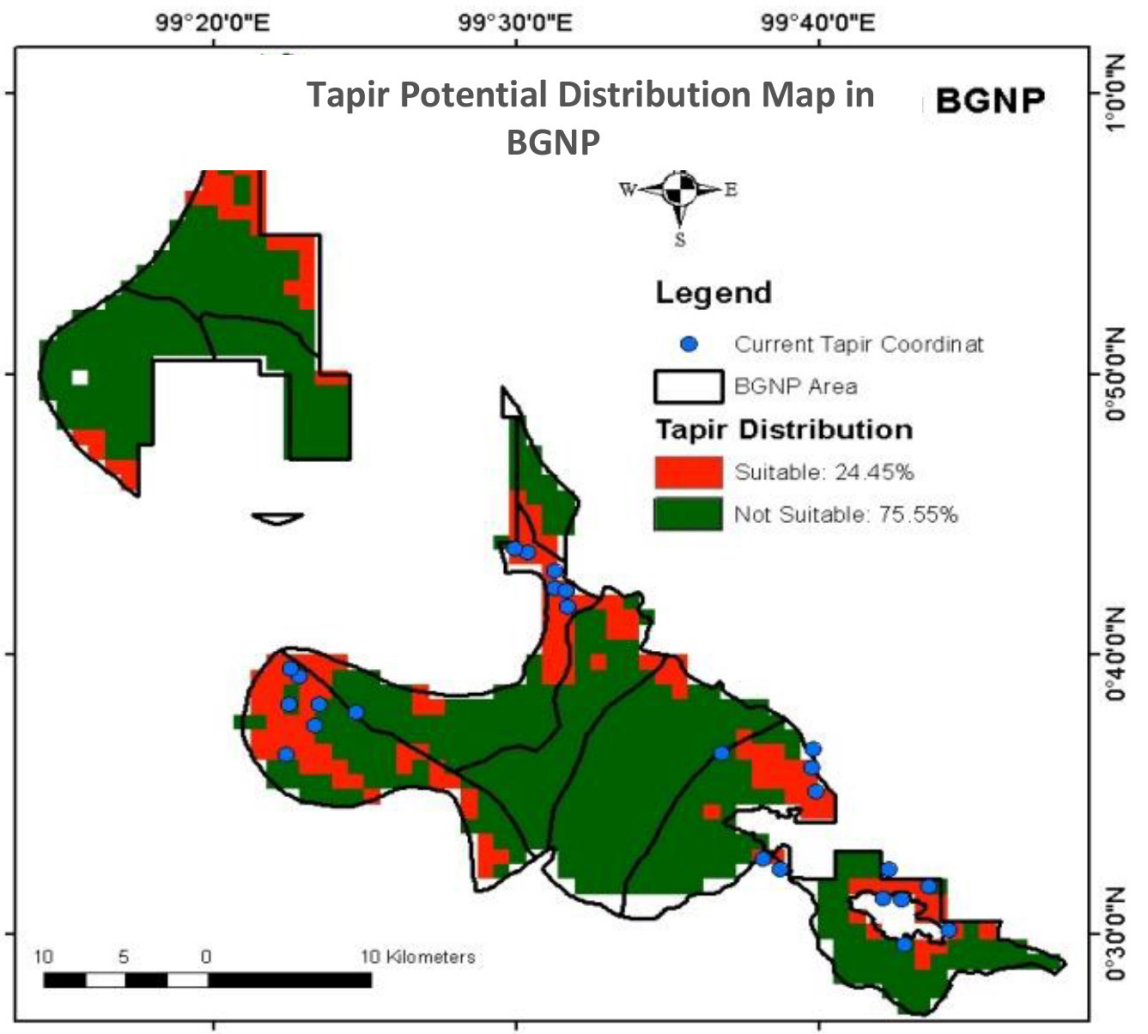
Tapir potential distribution map in BGNP.

Our modelling estimated an AUC of 0.869, indicating a good validity and reliability of the model to predict the distribution of tapirs in BGNP (Hijmans & Elith 2013; Phillips *et al*. 2006). The binary map estimated that 24.45% of the BGNP area is suitable for tapir habitat (Fig. 4). The model also showed that tapir habitats are generally near the border of BGNP. This finding is slightly identical to Schank *et al*. (2015) who modelled that only 27.2% of tapir suitable habitat across the Mesoamerican countries (except El Salvador) is in protected forests. In general, tapir habitats comprise PFs, SFs, mixed forests, rubber and oil palm plantations, community lands, and locations around human settlements (Gemita *et al*. 2007).

Among variables used to develop the model, zonal classification and bioclimatic factors (Bio 4 and Bio 13) have higher contributions to the model. This finding is identical to Schank *et al*. (2015) who also found that temperature and precipitation are variables with the greatest percentage contribution to the species distribution model for tapir across the Mesoamerican countries. According to Oliveira-Santos *et al*. (2010), temperature and rainfall are among the important variables for tapirs to select their habitats.

**Figure 5:**
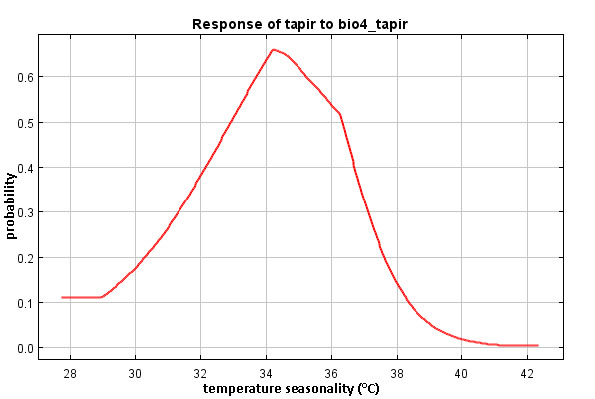
Response of tapir distribution model to the temperature seasonality.

Temperature seasonality (Bio 4) contributed 36.5% to the model. The model revealed that tapirs can be found in an area with a temperature around 32°C–7°C (the probability is greater than 0.4) (Fig. 5). The model also showed that the probability of tapir occurrence in BGNP will be lower when the temperature is above 37°C or less than 32°C. This finding is in contrast with Clements *et al*. (2012) who found a lower probability of tapir occurrence in Peninsular Malaysia in the range of temperatures greater than 25°C–26°C.

Association of Zoos and Aquariums (AZA) Tapir TAG (2013) pointed out that tapirs are heat tolerant that can tolerate temperatures up to 38°C. However, to maintain the thermal and water balance, tapirs alter their activity by becoming more nocturnal during the hotter days and diurnal during the colder days or select shaded and humid environments (Oliveira-Santos *et al*. 2010).

**Figure 6:**
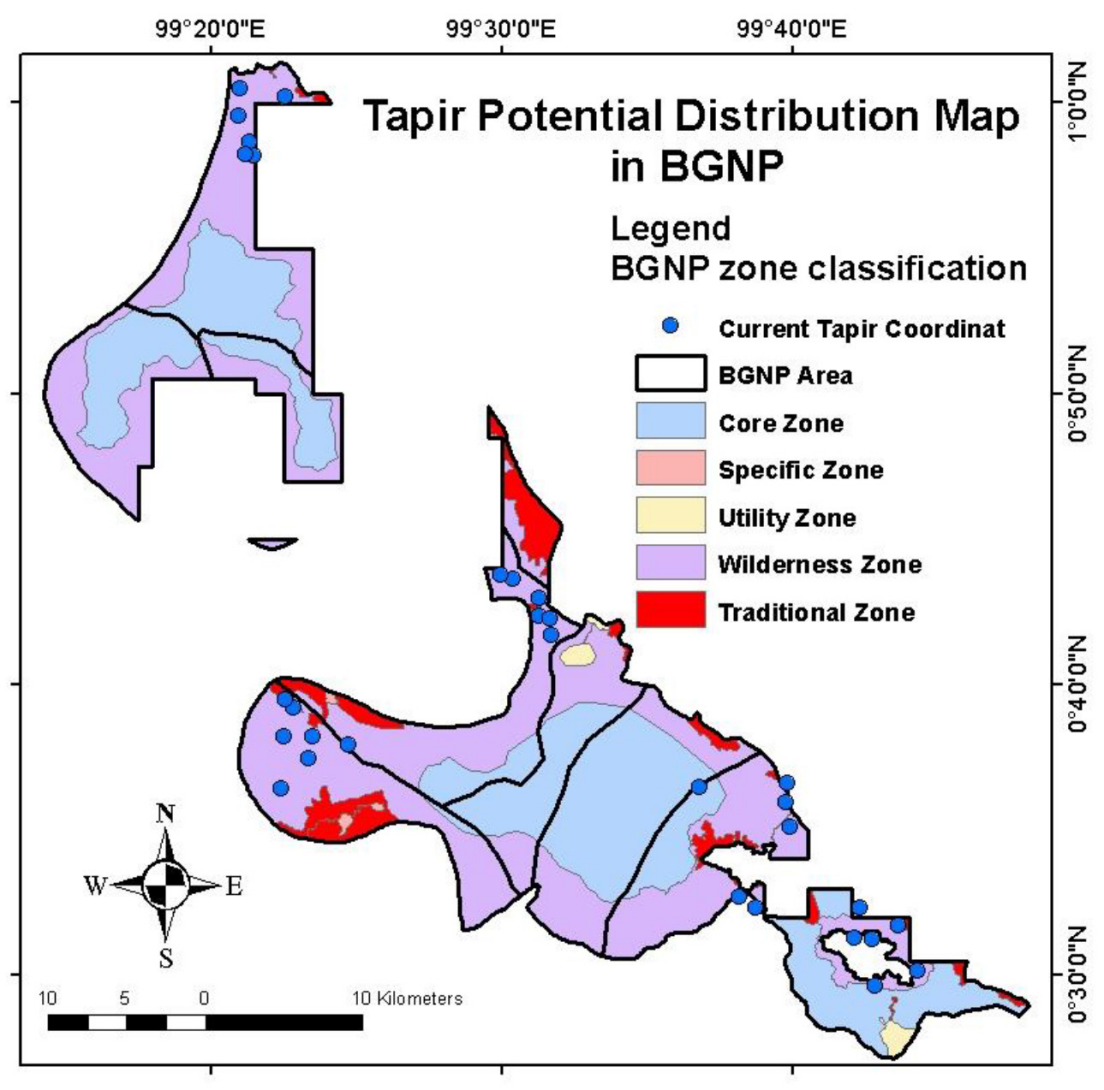
The tapir potential distribution model according to the zone in BGNP.

About 32.7% of the tapir distribution model in BGNP is explained by the zonal classification. Our modelling predicted the highest probability of tapir occurrence in the wilderness zone and a small part of the traditional zone (Fig. 6). Most of the wilderness and traditional zones adjacent to the buffer zone were used to be community gardens before being designated as a national park (Kuswanda & Antoko 2008). During our observation, these gardens have become OFs grown by numerous plants including tapir feeds plants. This condition is assumed to be one of the main drivers for tapirs to distribute in the wilderness and traditional zones.

**Figure 7:**
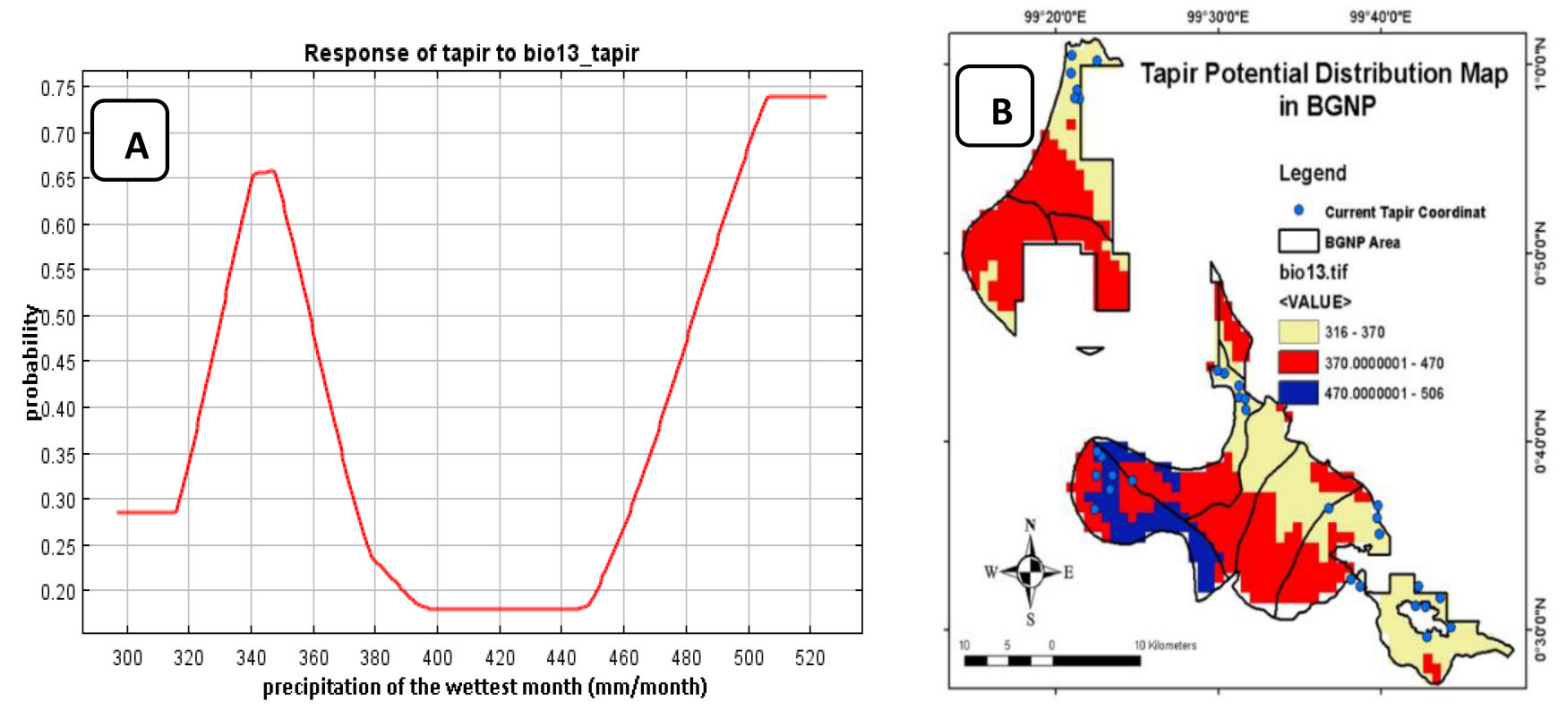
(A) Response of tapir distribution model to Bio 13; and (B) tapir potential distribution model based on precipitation.

Precipitation of the wettest month (Bio 13), which contributed 10.2% to the model, estimated a higher probability of tapir occurrence in the range of precipitation of 320 mm/month–370 mm/month and more than 470 mm/month (Fig. 7A). BGNP topographical conditions are ranging from lowlands to mountains. Different latitudes are expected to have different precipitations. The model also predicted that tapirs can be found in an area with the precipitations of 370 mm/month–470 mm/month, though the probability of occurrence is relatively low (Fig. 7B). Oliveira-Santos *et al*. (2010) pointed out that high rate of precipitation lead tapirs to be active throughout the day, to keep the thermal balance.

### The Characteristics of Tapir Habitat

#### Land use change in BGNP

Forest conversion may lead to habitat destruction and fragmentation, thereby threatening wildlife, including tapirs (Corlett 2007; Davis *et al*. 2013; Noga *et al*. 2018). Human activities in forests and hunting activities might also bring negative effects on wildlife, especially tapirs, as they reduce the active hours of tapirs and decrease the tapir population (Marlius *et al*. 2018; Ripple *et al*. 2016). Therefore, habitat monitoring is important in conserving tapirs.

This study found that forests in BGNP declined by more than 2.97% between 2000 to 2010 (Table 5). Surprisingly, from 2010 to 2020, forest covers increased by 3.07%, indicating crops and shrubs conversion into forests (Table 5).

#### Species diversity and vegetation composition in BGNP

This study found that land cover changes had a great impact on the characteristics of vegetation in BGNP. This finding is in accordance with previous studies stating that vegetation characteristics are highly affected by the land cover (Alikodra 2019; Borisade *et al*. 2021; Garizábal-Carmona & Mancera-Rodríguez 2021). Except for sapling in Sopotinjak OFs, the density and frequency of vegetation at various growth levels in SFs and OFs were higher than those of PFs (Table 6). Forest cover loss has allowed the sunlight to penetrate the forest floor and attracted pioneer species to grow (Dupuy & Chazdon 2008; Kuswanda & Mukhtar 2010). In PFs, only a certain species could adapt to high humidity, low temperature and wet forest floors (Fachrul 2007; Luo *et al*. 2021).

H’ of the vegetation at different growth stages in Pagar Gunung and Sopotinjak Resorts were classified as medium (2 ≤ H’ < 3) and high (H’ ≥ 3), respectively (Barbour *et al*. 1987). H’ of the seedling and understory in SFs and OFs is higher than PFs (Table 6). H’ of the sapling in Pagar Gunung SFs was higher than PFs. In Sopotinjak, H’ of the sapling for OFs was lower than that of PFs. In general, the H’ of seedling, understory, and sapling in all land covers was higher than those of natural forests in the Tesso Nillo National Park (TNNP). Kusumo *et al*. (2016) reported that the H’ of the seedling and sapling in natural forests of the TNNP were only about 2.18 and 2.05, respectively.

This study also indicates that the characteristics of vegetation are influenced by elevation. Species density and H’ of PFs in Sopotinjak (1,200 m) were higher than those of Pagar Gunung (1,400 m). Different elevations may have different weathers and microclimates that may affect species composition (Lu *et al*. 2021). This condition may also influence habitat carrying capacity, wildlife distribution, activities, and even its metabolic processes (Fuller *et al*. 2019; Kuswanda *et al*. 2021).

#### Important Value Index (IVI)

The IVI is a parameter used to measure the ecological significance of a species in a community or a site (Yuningsih *et al*. 2021). The number of plant species recorded in Pagar Gunung and Sopotinjak Resorts at the seedling, understory and sapling stages was 94. Pagar Gunung was occupied by 60 plant species (31 species in PFs and 39 species in SFs). In the seedling and understory stages, PFs and SFs were dominated by *R. teysmannii* and *Syzygium* sp., respectively (Table 7). In the sapling stage, PFs and SFs were dominated by *H. buruensis* and *P. obovatum*, respectively.

The total plant species in Sopotinjak was 75 (44 species in PFs and 43 species in OFs). PFs in Sopotinjak were dominated by *H. buruensis* (seedling and understory) and *L. brachystachys* (sapling). Meanwhile, OFs were dominated by *L. hexandra* (seedling and understory) and *P. sarmentosum* (sapling) (Table 7). Sambas *et al*. (2018) pointed out that the species with the highest IVI will become important species in the vegetation in the future.

### Tapir Feed Plants

Tapirs are selective browsers that choose high-quality plants for their feed (Gearty 2012). Tapirs spend most of their time foraging (Naranjo 2009). They forage in their home ranges and will return to the same location within 90 to 100 days (Maharani *et al*. 2019). Tapirs generally consume soft, fresh and easily chewed young leaves, fruits and new growth twigs (Holden *et al*. 2003; Williams & Petrides 1980). They also consume grasses and pioneer species, including *Artocarpus* sp. (Moraceae). In total, tapirs feed on at least 380 plant species (Simpson *et al*. 2013).

**Figure 8:**
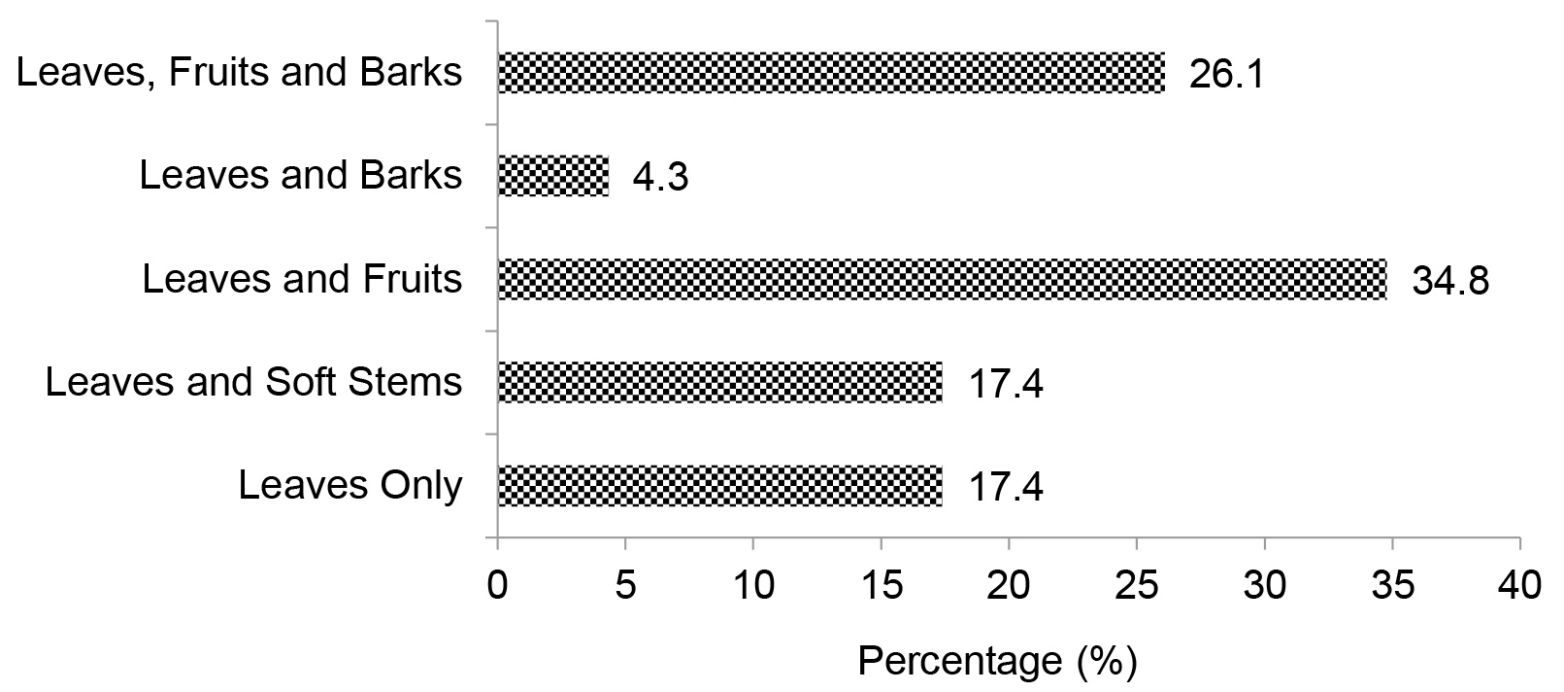
The percentage of plant parts consumed by tapir.

During this study, about 23 feed plant species (14 families) were identified in Pagar Gunung and Sopotinjak Resorts (see Appendix). These feeds are dominated by the Moraceae family. These feeds were generally consumed for their leaves (Fig. 8). About 34% of the feeds were also consumed for their fruits. About 26.1% of feeds, e.g., *Artocarpus elasticus*, *Ficus toxicaria* and *Pouzolzia zeylanica*, were consumed entirely by tapirs. This finding is identical to Tobler *et al*. (2006) who also found that the dominant plant parts consumed by tapirs are leaves. In general, the number of leaves eaten by tapirs varies between feed plant species (Simpson *et al*. 2013). Tapirs prefer nitrogen-fixing species (Downer 2001). The daily feed consumption of tapirs is about 4%–5% of its body weight (Gearty 2012).

**Figure 9:**
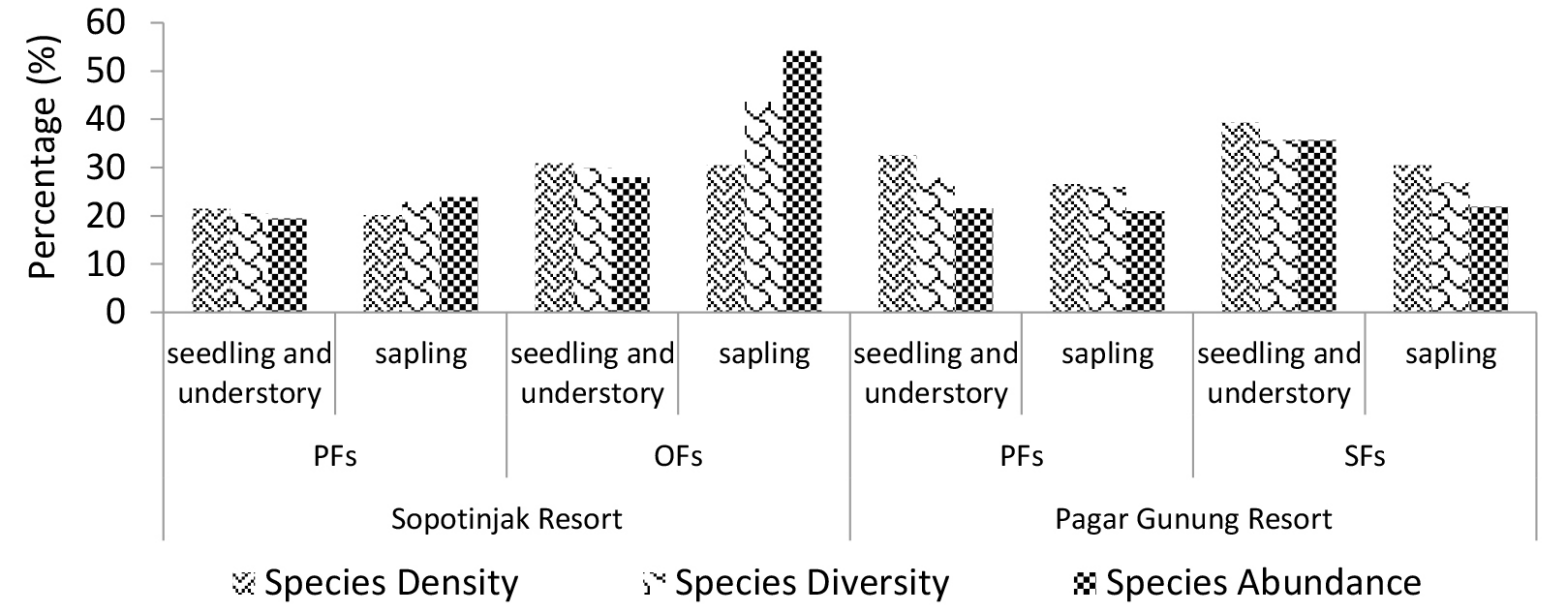
The characteristic of feeds in different resorts and growth stages.

The proportion of feed plants to the total vegetation in Pagar Gunung and Sopotinjak Resorts was less than 40%. The density, diversity and abundance of feed plants at the seedling, understory and sapling stages in OFs and SFs were higher than in PFs (Fig. 9). The high density, diversity and abundance of feed in SFs and OFs may attract the tapir to browse feed in SFs and OFs. In general, tapirs prefer to forage in SFs because SFs are grown with numerous young plants stimulated by sunlight penetration on the forest floor (Samantha *et al*. 2020; Schank *et al*. 2020; Tobler 2002). Moreover, feeds grown in SFs have high concentrations of proteins and nutrients in their buds and leaves (Salas & Fuller 1996).

A chi-square analysis revealed that the asymp. sig. (2-sided) value was 0.245 (> 0.05), indicating there is no relationship between vegetation characteristics and tapir feed plants in each type of land cover for various growth stages. This finding is identical to Salas and Fuller (1996) who also found that there was no relationship between the abundance of plant species and the abundance of tapir feed plants in Tabaro River valley, Southern Venezuela.

### Implications for Tapir Conservation Programmes

Tapirs require a huge area for foraging (Marlius *et al*. 2018). Tapirs generally prefer a highly vegetated habitat with a dense canopy cover, lots of food and water, and far from roads (Samantha *et al*. 2020). This study clearly showed that the tapir habitat in BGNP is still in good condition with low forest disturbances in the last 10 years. The tapir habitat in BGNP was also occupied by various plants that can be used as food for tapirs. However, concerns should be given to the distribution of tapirs in BGNP. The model showed that tapirs distribute near the traditional zone where community crops are growing. This condition may attract tapirs to enter the gardens, destroy the crops and generate human-tapir conflicts. Previous studies have shown that tapirs prefer to consume community crops, e.g., young bean shoots, corn, cabbage, pineapple, potatoes and watermelon (Novarino 2005; Suárez & Lizcano 2002; Waters 2015). Local people also stated that tapirs also prefer to consume coffee and pumpkin planted by local people in BGNP.

Our interview with local communities revealed that tapir foot marks were frequently observed in the garden, indicating that tapirs in BGNP have started to invade the community garden. At this stage, local people still have a good perception of tapirs, and there are no records of human-tapir conflicts or tapirs hunting in BGNP surrounding villages. When conflict intensity is escalating, however, people’s perceptions and attitudes toward tapirs may change, and human-tapir conflicts may occur in this national park.

This issue is needed to consider by the BGNP management in managing tapir conservation programmes. Without management intervention, human-tapir conflict may occur, and the population of tapir in BGNP will continue to decline, as experienced by the Tapanuli orangutan (Kuswanda *et al*. 2021).

We suggest the BGNP management to perform the following strategies:

1. Enriching OFs of wilderness and traditional zones with feed plants which are not utilised by local communities, such as *Artocarpus elasticus, Ficus toxicaria*, *Mikania scandens, Hemigraphis buruensis* and *Zingiber* sp.
2. Trimming the tree crown in the wilderness and utilisation zones to accelerate the growth of feed plants on the forest floor.
3. Providing education, training and guidance for local people to live harmoniously with tapirs.
4. Involving local community in tapir conservation programmes.
5. Encouraging community to grow non-palatable crops for tapirs.
6. Providing compensation scheme for local people affected by tapirs.

## CONCLUSION

The most important variables in developing the species distribution model for tapir in BGNP are zonal classification, temperature and precipitation. Our modelling reveals that 24.45% of BGNP areas are suitable for tapir habitat. We also found that tapir habitat conditions in BGNP are still in a good condition where forest conversion was absent in the last 10 years. About 23 feed plant species dominated by the Moraceae family were identified in Pagar Gunung and Sopotinjak Resorts. These feeds were predominantly consumed for their leaves. About 34% of feeds were also consumed for their fruits, and 26.1% of the feeds were consumed entirely by the tapir. The model also shows that tapirs distribute near the traditional zone where the community garden is located. This condition may attract tapirs to have close contact with humans, and lead to human-tapir conflicts. BGNP management needs to consider this finding in developing tapir conservation programmes in BGNP. Several strategies proposed to overcome this issue are planting feed plants in Ofs, e.g., the wilderness and traditional zones; reducing the canopy cover in the wilderness and utilisation zones to enhance the growth of feed plants; educating, training and guiding local people to live harmoniously with tapirs; engaging the community in tapir conservation programmes; suggesting the community to cultivate non-preferred crops by tapirs; and providing compensation scheme for farmers disturbed by tapirs.

## Figures and Tables

**Table 1:** Environmental variables used to develop SDM for tapir in BGNP.

No	Environmental variables	Source	Type	Extraction methods
1	Elevation	ASTER GDEM (https://earthexplorer.usgs.gov/)	.tif	Spatial analysis
2	Slope	Spatial analysis from elevation data	.tif	Analysis of the slope from surface topography
3	Climate	www.worldclime.org	.bil	Spatial analysis (Bio 1, 5, 6, 12, 13, 14)
4	BGNP zone	BGNP	.shp	Spatial analysis
5	Land cover	Landsat Image 8 (https://earthexplorer.usgs.gov/)	.tif	Spatial analysis (year 2020)

**Table 2:** Bioclimatic predictors used in developing species distribution model for tapir in BGNP.

No	Bioclimatic predictors	Explanation
1	Bio 1	Annual mean temperature (°C)
2	Bio 4	Temperature seasonality (°C)
3	Bio 5	Max temperature of the warmest month (°C)
4	Bio 6	Min temperature of the coldest month (°C)
5	Bio 7	Annual temperature range (°C)
6	Bio 12	Annual precipitation (mm/month)
7	Bio 13	Precipitation of the wettest month (mm/month)
8	Bio 14	Precipitation of the driest month (mm/month)
9	Bio 15	Precipitation seasonality (mm/month)

**Table 3:** Zonal classification of BGNP.

No	Zone/*Zona*	Total area (Ha)	Explanation
1	Core zone (*Zona inti*)	28,281.73	Non-use areas (research purposes only)
2	Specific zone ( *Zona khusus*)	169.22	Use for specific purposes, e.g., cultural preservation
3	Utility zone (*Zona pemanfaatan*)	665.61	Areas for other purposes, such as ecotourism and non-timber forest products
4	Wilderness zone (*Zona rimba*)	38,870.43	Forest areas with limited utilisation (research, education, special tourism); a buffer of the core zone
5	Traditional zone (*Zona tradisional*)	4,816.76	Areas for the economic development of traditional communities

**Table 4:** Land cover classification in BGNP.

No	Land cover	Notes
1	Crops	Consists of crops and rice plants
2	Forests	Consists of primary and secondary forests
3	Open areas	Non vegetation area
4	Residential areas	Human settlements
5	Shrubs	Consists of shrubs and juvenile plants

**Table 5:** Land-cover change in BGNP from 1990 to 2020.

Class	Percentage of area per year (%)				Change (%)			

	1990	2000	2010	2020	1990–2000	2000–2010	2010–2020	1990–2020
Forest	91.36	91.36	88.39	91.46	0	−2.97	+3.07	+0.1
Crop	4.32	4.32	4.59	3.72	0	+0.27	−0.87	−0.60
Shrub	4.18	4.18	6.75	4.55	0	+2.57	−2.20	+0.37
Residental	0.14	0.14	0.14	0.14	0	0.00	0.00	0.00
Open Area	0	0	0.13	0.13	0	+0.13	0.00	+0.13

*Note*: The change reveals the increase or the decrease of a land cover in a certain period. The “+” and “− “signs represent the increase and the decrease of the land cover, respectively.

**Table 6:** The density and diversity of vegetation around the tapir habitat in BGNP.

Resort	Land cover	Growth stage	D	F	H’	N
Sopotinjak	PFs	Seedling and understory	51,250.00	8.40	3.15	34.36
		Sapling	3,760.00	6.30	3.07	28.30
	OFs	Seedling and understory	65,500.00	9.60	3.34	39.55
		Sapling	2,880.00	4.00	2.53	18.79

Pagar Gunung	PFs	Seedling and understory	71,470.59	6.35	2.66	26.91
		Sapling	5,035.29	3.71	2.34	17.60
	SFs	Seedling and understory	78,875.00	11.75	3.16	31.37
		Sapling	5,840.00	7.40	2.78	22.03

*Notes*: D = density; F = frequency, H’= h-index (the Shannon-Wiener diversity index); N = the abundance index.

**Table 7:** Top three dominant species in different land cover in different resorts.

Research site	Land cover	Growth stage	Scientific name	RD (%)	RF (%)	IVI (%)
Pagar Gunung	PFs	Seedling and understory	*Rhodoleia teysmannii* Mig	18.11	14.82	32.92
			*Syzygium* sp.	16.05	12.96	29.01
			*Hemigraphis buruensis* Hall.f	13.79	10.19	23.97
		Sapling	*Syzygium* sp.	2.9	19.03	41.92
			*Litsea brachystachys* Boerl.	24.77	15.86	40.62
			*Palaquium obovatum* Engl., var.	16.82	15.86	32.68
	SFs	Seedling and understory	*Hemigraphis buruensis* Hall.f.	12.36	6.81	19.17
			*Litsea brachystachys* Boerl.	6.50	7.23	13.73
			*Syzygium* sp.	5.71	6.81	12.51
		Sapling	*Palaquium obovatum* Engl., var.	14.04	11.49	25.53
			*Syzygium* sp.	13.01	10.81	23.82
			*Litsea brachystachys* Boerl.	10.62	11.49	22.10
Sopotinjak	PFs	Seedling and understory	*Hemigraphis buruensis* Hall.f.	18.92	11.90	30.83
			*Elatostema lineolatum* Wight	8.25	5.95	14.20
			*Diplazium proliferum* Thouash	4.85	7.14	12.00
		Sapling	*Litsea brachystachys* Boerl.	12.77	9.52	22.29
			*Litsea odorifera* Valeton	7.45	9.52	16.97
			*Syzygium* sp.	7.45	7.94	15.38
	OFs	Seedling and understory	*Lameta hexandra* Swartz	11.83	7.29	19.12
			*Pluchea indica* L.	6.87	6.25	13.12
			*Hemigraphis buruensis* Hall.f.	7.25	5.21	12.46
		Sapling	*Piper sarmentosum* Roxb. ex.Hunter	19.44	12.50	31.94
			*Cinnamomum burmani* (C.G. & Th. Nees) Nees ex Blume	13.89	12.50	26.39
			*Saurauia pendula* Blume	13.89	12.50	26.39

*Notes*: RD = Relative Density, RF = Relative Frequency

## APPENDIX

### Appendix A The Asian tapir feed plants in BGNP

[Table t8-tlsr-34-2-57]

**Table t8-tlsr-34-2-57:**

No	Scientific name	Family	Plant parts consumed by Asian tapir
1	*Aglaia argentea* Blume	Meliaceae	Leaves
2	*Artocarpus elasticus* Reinw.	Moraceae	Leaves, fruit and bark
3	*Artocarpus heterophyllus* Lamk.	Moraceae	Leaves, fruit and bark
4	*Artocarpus integler* (Thunb). Merr.	Moraceae	Leaves, fruit and bark
5	*Artocarpus rigidus* Blume	Moraceae	Leaves, fruit and bark
6	*Begonia isoptera* Dryyand.ex.J.E Smith	Begoniaceae	Leaves and fruit
7	*Cucurbita moschata* Durch	Cucurbitaceae	Leaves and fruit
8	*Elatostema lineolatum* Wight	Urticaceae	Leaves and soft stem
9	*Eurea acuminata* A.P.DC.	Theaceae	Leaves and bark
10	*Ficus drupacea* Thunb.	Moraceae	Leaves and fruit
11	*Ficus glandulifera* Wall	Moraceae	Leaves and fruit
12	*Ficus toxicaria* Linn.	Moraceae	Leaves, fruit and bark
13	*Hemigraphis buruensis* Hall.f.	Acanthaceae	Leaves and soft stem
14	*Ixonanthes petiolaris* Blume	Linnaceae	Leaves and fruit
15	*Litsea velutina* Boerl.	Lauraceae	Leaves
16	*Mikania scandens* Willd.	Asteraceae	Leaves and soft stem
17	*Palaquium obovatum* Engl., var.	Sapotaceae	Leaves
18	*Phyllanthus indicus* Muell. Arg.	Euphorbiaceae	Leaves
19	*Plectronia indica* Linn.	Rubiaceae	Leaves and fruit
20	*Pouzolzia zeylanica* Benn.	Urticaceae	Leaves, fruit and bark
21	*Schefflera aromatica* (Blume) Harms	Araliaceae	Leaves and soft stem
22	*Sechium edule* (Jacq.) Swartz	Cucurbitaceae	Leaves and fruit
23	*Zingiber officinale* Rosc	Zingiberaceae	Leaves and fruit
